# Scavenger Receptor A Mediates the Clearance and Immunological Screening of MDA-Modified Antigen by M2-Type Macrophages

**DOI:** 10.1007/s12017-017-8461-y

**Published:** 2017-08-21

**Authors:** Andreas Warnecke, Sonja Abele, Sravani Musunuri, Jonas Bergquist, Robert A. Harris

**Affiliations:** 1 Applied Immunology and Immunotherapy, Department of Clinical Neuroscience, Karolinska Institutet, Center for Molecular Medicine, Karolinska University Hospital at Solna, 17176 Stockholm, Sweden; 2 Department of Chemistry-BMC, Analytical Chemistry and Science for Life Laboratory, Uppsala University, Box 599, 751 24 Uppsala, Sweden

**Keywords:** Malondialdeyde, Macrophages, MOG-EAE, Autoimmunity, Scavenger receptors, Posttranslational modifications

## Abstract

In this study, we investigated the uptake of malondialdehyde (MDA)-modified myelin oligodendrocyte glycoprotein (MOG) in the context of lipid peroxidation and its implications in CNS autoimmunity. The use of custom-produced fluorescently labeled versions of MOG or MDA-modified MOG enabled us to study and quantify the uptake by different macrophage populations and to identify the responsible receptor, namely SRA. The SRA-mediated uptake of MDA-modified MOG is roughly tenfold more efficient compared to that of the native form. Notably, this uptake is most strongly associated with anti-inflammatory M2-type macrophages. MDA-modified MOG was demonstrated to be resistant to degradation by lysine-dependent proteases in vitro, but the overall digestion fragments appeared to be similar in cell lysates, although their relative abundance appeared to be altered as a result of faster uptake. Accordingly, MDA-modified MOG is processed for presentation by APCs, allowing maximized recall proliferation of MOG_35-55_-specific 2D2 T cells in vitro due to higher uptake. However, MDA modification of MOG did not enhance immune priming or disease course in the in vivo MOG-EAE model, but did induce antibody responses to both MOG and MDA adducts. Taken together our results indicate that MDA adducts primarily constitute clearance signals for phagocytes and promote rapid removal of antigen, which is subjected to immunological screening by previously licensed T cells.

**Electronic supplementary material:**

The online version of this article (doi:10.1007/s12017-017-8461-y) contains supplementary material, which is available to authorized users.

## Introduction

### MDA Adducts in the Context of Autoimmunity

Malondialdehyde (MDA = 1,3-propionaldehyde) is a highly reactive three-carbon dialdehyde generated as a breakdown product from peroxidized lipids, among other reactive aldehydes (reviewed in Nam 2011; Papac-Milicevic et al. 2016; Busch and Binder 2017). Specifically, MDA is also an established by-product from the synthesis of thromboxanes and prostaglandin metabolism (Nam 2011; Papac-Milicevic et al. 2016; Busch and Binder 2017; Hamelin and Chan 1983; Hammarström and Falardeau 1977). Together the major sources of MDA associate its generation to oxidative stress and inflammation.

MDA reacts with primary ε-amines on lysines in proteins to form a range of adducts and cross-links as described in more detail elsewhere (Slatter et al. 1998; Tuma et al. 2001). The predominant reaction products are single MDA adducts and more advanced malondialdehyde-acetaldehyde (MAA) adducts that involve the formation of a dihydropyridine ring (Antoniak et al. 2015). The latter is deemed more stable due to its resistance of spontaneous hydrolysis, and its generation is enhanced in the presence of aldehydes (Mooradian et al. 2001) that may derive from breakdown of MDA itself (Slatter et al. 1998) or from exogenous sources (McCaskill et al. 2011; Freeman et al. 2005).

Advanced lipoperoxidation products (ALE) are specifically recognized by components of innate immunity (Harris and Amor 2011; Weismann and Binder 2012). In this context, MDA adducts have previously been reported to enhance immune responses to its carrier protein and to evoke a range of pro-inflammatory mechanisms (Wallberg et al. 2007; Wuttge et al. 1999; Willis et al. 2002; Veneskoski et al. 2011; Höhn et al. 2014; Willis et al. 2004; Ott et al. 2014; Duryee et al. 2004; Thiele et al. 1998). These pro-inflammatory features motivate investigation of MDA adducts as potential triggers of autoimmunity (Anderton 2004; Doyle and Mamula 2005; Warnecke et al. 2014).

### Multiple Sclerosis and MOG as a Model Antigen

With this in mind, investigating MDA/MAA adducts in the context of immunology has become especially interesting to address because: (1) recent studies imply the generation of MDA adducts in lungs of smokers (McCaskill et al. 2011; Freeman et al. 2005); (2) the lungs have been proposed as an organ in which CNS-reactive T cells are licensed (Odoardi et al. 2012); and (3) smoking is a well-established risk factor for autoimmune diseases (Perricone et al. 2016). Specifically for multiple sclerosis (Dendrou et al. 2015), there is independent evidence for elevated lipid peroxidation and breakdown products thereof in plasma and CSF of patients (Ferretti and Bacchetti 2011; Gonzalo et al. 2012; Miller et al. 2011), warranting the investigation of MDA and its impact on the antigenicity of established MS autoantigens such as myelin oligodendrocyte glycoprotein (MOG). MOG is a CNS-specific autoantigen expressed on the surface of mature oligodendrocytes and is targeted during the autoimmune CNS inflammation characteristic for MS (Dendrou et al. 2015; Breithaupt et al. 2003; Clements et al. 2003). The localization of MOG on the cell surface makes it a prime target for damage related to products of lipid peroxidation such as MDA. Furthermore, MOG is a well-established autoantigen widely used to evoke the animal model of MS, namely experimental autoimmune encephalomyelitis (EAE), with infiltrating monocytes being the major cause of pathogenesis (Simmons et al. 2013; McCarthy et al. 2012).

### Scavenger Receptor A

Scavenger receptor A (SRA) is a member of the scavenger receptor family of pattern recognition receptors. Structurally SRA belongs to class A scavenger receptors and features only a short cytosolic tail followed by a single transmembrane domain, a coiled coil, two collagen-like domains and a scavenger receptor cysteine-rich domain (SRCR) (Canton et al. 2013). The functional complex exists as an SRA trimmer (Platt et al. 2002) and mediates interaction with a variety of different ligands via a combination of topology and charge interactions involving its extracellular domains (Canton et al. 2013; Platt et al. 2002). The cellular expression of SRA is strongly associated with macrophages and monocyte-derived APCs (cf: www.immgen.org), reflected by its synonym macrophage scavenger receptor 1 (MSR1). Functionally, SRA mediates the phagocytosis of cognate patterns present on bacterial surfaces or in the form of modified self-antigens (Canton et al. 2013; Platt et al. 2002; Berger et al. 2014). This receptor-mediated phagocytosis is highly efficient in clearing bacterial load or debris but can also result in pathological situations if the macrophage is overwhelmed, as is the case for lipid-laden ‘foam cells’ in atherosclerosis (Canton et al. 2013). Generally, the expression of scavenger receptors shapes the functional phenotypes of either pro-inflammatory (M1) or alternatively activated (M2) polarized macrophages by modulating inflammatory or homeostatic responses (Canton et al. 2013; Mia et al. 2014).

### Study Outline

Custom-made fluorescently labeled versions of MOG or MDA-modified MOG enabled us to study and quantify the uptake by different macrophage populations and to screen for the responsible receptor, identified as SRA. The experiments further investigate the uptake, digestion and immunogenicity of the of MDA-modified MOG compared to the native form in vitro and in vivo.

Notably, highly efficient uptake mediated by SRA is most strongly associated with anti-inflammatory M2-type macrophages. While in vitro assays establish a higher uptake and stimulation of MOG_35-55_-specific 2D2 T cells with MDA-modified MOG, active immunization in the EAE animal model displayed no impact on disease course or severity. Interestingly, the immunization with MDA-modified MOG did induce antibody responses to both MOG and the MDA adducts. Collectively our data imply that MDA adducts primarily constitute clearance signals for phagocytes and promote rapid removal of antigen, but likewise that this cargo is subject to immunological screening by previously licensed T cells.

## Materials and Methods

### Recombinant Mouse MOG (MOG) Production and Modification

HIS-tagged mouse MOG was purified from *E.* *coli* as previously described (Warnecke et al. 2017). Fluorescent labeling was performed by reacting freshly DMF-dissolved Dylight650-4xPEG NHS Ester (Thermo Scientific™) at a molar ratio of 1:2.9 (MOG:Dylight) for 1 h at room temperature. Non-reacted dye was removed by dialysis against PBS (3500 MWCO, 4 °C, at least 4 complete buffer exchanges, min. 24 h total dialysis time). Protein concentrations were determined using the BCA assay kit (Thermo Scientific™). Absorbance spectra were measured for 350–800 nm, and the value at 660 nm was used to calculate the degree of labeling. The procedure was optimized to yield a final 1:1 ratio. The fluorescent properties were measured using a GloMax^®^-Multi + Microplate Multimode Reader with Instinct^®^ software using a filter for excitation at 625 nm and emission at 660 nm. Throughout the manuscript, the Dylight650-4xPEG dye will be abbreviated as ‘Dy.’

MDA was prepared by acid hydrolysis of 1,1,3,3-tetramethoxypropane (Aldrich). The hydrolyzed MDA solution was neutralized by addition of 4× concentrated PBS, MilliQ and 1 M NaOH for titration to neutral pH (0.5 M stock). Aliquots of MOG or MOG-Dy were modified by adding 200 µL/mL (~50 mM final) of the pH neutral MDA and additionally acetaldehyde at a concentration of 2.8 µL/mL (~25 mM final) to promote the generation of MAA adducts (Mooradian et al. 2001). The reaction was incubated >1 day at 37 °C. Non-reacted MDA was removed from protein by dialysis against PBS as described above. Despite promoting the generation of MAA adducts, we refer to the MDA/MAA-modified MOG collectively as MOG-MDA in this manuscript (cf. Supplementary Fig. 1).

### Biochemical Analyses

SDS-PAGE, isoelectric focusing, Coomassie staining and Western blotting were performed as previously described (Warnecke et al. 2017). Protein digests or cell lysates were separated using Novex^®^ Tricine gels. Cell lysates were obtained by lysis of washed cells on ice using NP40-containing RIPA buffer (Sigma) supplemented with Complete^®^ protease inhibitor cocktail (Roche) and cleared by centrifugation (4 °C, 30 min, 13 000 rpm). Protein content was determined using the BCA assay kit (Thermo Scientific™) and up to 30 µg used as input for SDS-PAGE.

Primary antibodies for Western blotting are: 8-18C5 anti-MOG (mouse monoclonal, in-house production), anti-HIS-tag (mouse monoclonal, BioRad/Serotec MCA1396), anti-MDA serum (rabbit polyclonal, Alpha Diagnostic MDA11-S), anti-MDA/MAA 1F83 (mouse monoclonal), anti-actin (rabbit polyclonal, Sigma A2066). Detection of Dy-labeled protein from Western membranes or directly from SDS-PAGE gels (offset = 0.5 mm) was performed using the Li-COR Odyssey^®^ CLx system scanning both channels (700 and 800 nm).

### In Vitro MOG Digestion

All digestion enzymes were purchased from Sigma and used at their pH optimum and recommended dose (~5% w/w) according to the manufacturer’s information. Enzymes are: Cathepsin B from human placenta (C0150), Cathepsin C from bovine spleen (C8511), Cathepsin D from bovine spleen (C3138), Cathepsin L from human Liver (C6854), endoproteinase Lys-C from *Lysobacter enzymogenes* (P3428), Trypsin from porcine pancreas (T6567).

### Cell Cultures and T Cell Proliferation Assays

RAW264.7 cells, splenocytes from MOG-reactive TCR transgenic 2D2 mice or lymphocytes from immunized wild-type C57BL/6 mice were cultured under standard conditions and assayed for proliferation using H^3^-methyl-thymidine incorporation as previously described (Warnecke et al. 2017).

Primary bone marrow-derived macrophage cultures from C57BL/6 mice or SRA^−/−^ mice (generously provided by the Mikael Karlsson Lab, Karolinska Institutet) were initiated by flushing femoral bones from using 21-gauge needles and washing the cells with PBS. Single cell suspensions were cultured in Dulbecco’s modified Eagles Medium (DMEM, Sigma) supplemented with 10% heat-inactivated fetal bovine serum (FBS, Sigma), 100 U/mL penicillin, 100 µg/mL streptomycin, 2 mM L–glutamine, 1 mM Sodium pyruvate and 20 µM β-mercaptoethanol (all reagents from Life Technologies) as well as 20 ng/mL of either M-CSF or GM-CSF (R&D Systems) for macrophage differentiation. The macrophages were allowed to differentiate for a total of 10 days in 175 cm^2^ green cell culture flasks (Sarstedt) 37 °C and 5% CO_2_, with a half change of medium at day 4 and a full change at day 6. Cells were re-plated after detachment using pre-warmed 2 mM EDTA in PBS (20 min incubation and scraping) and ensuing washes. Finally, induction of macrophage phenotype polarization to M0 (media alone, 24 h), M1 (50 ng/mL LPS, 100 ng/mL IFNγ, 24 h) or M2 (IL-4, IL-10 and TGF-β 20 ng/mL each, 24 h) was performed prior to the respective assays.

### FACS Antigen Uptake Assay and Intracellular Staining

RAW264.7 cells or bone marrow macrophages were seeded in 48 flat-bottom plates (Thermo Fischer Scientific) at 100,000 cells/well and allowed to rest or differentiate for 24 h. The wells were rinsed before applying fresh media with either MOG-Dy or MOG-Dy-MDA (10 µg/mL) for up to 4 h. Inhibitors or competing compounds (Sigma) were added 30 min prior to adding the protein. Blocking antibodies and isotype controls are: anti-SRA (MSR1) (20 µg/mL), anti-MARCO (20 µg/mL), anti-SRB1 (1 µg/mL), anti-CD36 (10 µg/mL), anti-goat IgG (20 µg/mL), rat IgG2a (10 µg/mL), rabbit serum (1 µg/mL). Blocking compounds: fucoidan, dextran sulfate, Mannan (all at 100 µg/mL). Small molecule inhibitors are: BLT-1 (SR-B1 small molecule antagonist), cytochalasin D 1 (actin polymerization antagonist) (both at 10 µM). Cells were washed and harvested using EDTA before transfer to 96-well V-bottom plates and stained using live/dead cell marker (Life Technologies) for 20 min at 4 °C. In some setups, media controls were additionally stained using anti-SRA (MSR1/CD204) antibodies (Biosite LS-C17770 or R&D Systems FAB1797A) at 0.5 µg/mL in 50 µL. Stained samples were washed twice and re-suspended in PBS before acquisition with a fluorescence activated cell sorting (FACS) Gallios flow cytometer (Beckman Coulter, Brea, CA, USA). Analysis was performed using Kaluza software (v. 1.3, Beckman Coulter).

Intracellular stainings of HIS-tag stainings in macrophages to assess uptake, or Ki-67 staining in T cells, were performed using reagents from BD Biosciences as follows: Extracellularly stained cells were thoroughly washed with PBS (300×*g*, 4 °C, 10 min) before fixation/permeabilization for >30 min at 4 °C. Fixed cells were washed with perm/wash buffer (400×*g*, 4 °C, 10 min) before staining (4 °C, 30 min, dark, perm/wash buffer as diluent). Unbound antibody was removed by repeated washed using per/wash buffer before re-suspending cell in PBS for acquisition.

Antibodies are: anti-HIS-tag (BioRad/Serotec MCA1396A647); anti-Ki-67 (BD Bioscienes); note that voltages were set per experiment for optimal sensitivity; thus, MFI values can only be compared within but not between independent experiments.

### RT-PCR and Heatmap

RT-PCR and heatmap generation were performed in principal as previously described (Mia et al. 2014).

### Animals and MOG-Induced Experimental Autoimmune Encephalomyelitis (EAE)

Animal experimentation conformed to Swedish legislation and was approved by the local ethical committee with permits N162/12 and N138/14. EAE immunizations were carried as previously described (Warnecke et al. 2017) using 25–50 µg of protein emulsified in complete Freund’s adjuvant containing 50 µg/100 µL *Mycobacterium tuberculosis*, along with intraperitoneal injections of 200 ng *pertussis* toxin at the time of immunization and 48 h later. The EAE severity score ranged from 0 (healthy) to 5 (death), but EAE severity typically peaked at score 3, corresponding to bilateral hind limb paralysis.

### Serum ELISA

Cardiac blood was sampled from freshly sacrificed EAE animals, allowed to coagulate and serum collected after centrifugation to be stored at −20 °C until use. Nunc Maxisorb™ flat-bottom plates were coated with 1 µg of protein/well in 0.1 M NaHCO_3_ pH 8.2 (100 µL/well, 4 °C, overnight). Wells were washed and subsequently blocked (5% BSA in PBS, 250 µL/well, 1 h, RT). Throughout the ELISA, 1% BSA in PBS was used as dilution buffer for sera or reagents. After 3 washes with 0.05% PBS-Tween20, the wells were incubated with diluted sera (1:5000, RT, 1 h, mild shaking). After additional washes, HRP-conjugated isotype antibodies were applied (Life Technologies: IgG/IgM: A-10677, IgG1: A10551, IgG2a: 61-0220; 1:2000, 1 h, RT, mild shaking). Plates were thoroughly washed 7× using 0.05% PBS-Tween20 before adding tetramethylbenzidine substrate solution (100µL TMB, Sigma) and development (RT, ~15 min, shaking). Color development was terminated identically for each isotype by addition of 100µL 2 N H_2_SO_4_. The OD values were calculated from the difference in absorbance at 450–570 nm.

### Statistics

Statistics were analyzed using GraphPad Prism v 5.04. Significance testing between two samples was performed using Student’s T Test. Grouped data were analyzed using ANOVA and Bonferroni-corrected multiple comparison posttests using an alpha of 0.05. Stars indicate p values as follows: * = *p* < 0.05, ** = *p* < 0.01, *** = *p* < 0.001, **** = *p* < 10^−4^. Bracketed stars indicate individually performed T tests where the Bonferroni-corrected test was not significant and represent low confidence comparisons.

## Results

### Production of Custom-Made MOG-Dylight650-MDA to Study Uptake

We employed fluorescent labeling to study the uptake of MDA-modified MOG (cf. Suppl. Fig. 1). This enabled us to use an array of methods to study uptake independently from other processes, such as degradation, and that circumvented many of the technical limitations associated with other approaches (cf. Suppl. Fig. 2). Our main interest was to study how posttranslational modification would affect antigen uptake. It was therefore not immediately relevant whether the dye itself would potentially affect the uptake of the antigen, but rather how MDA modification would, with the unmodified MOG serving as the reference control. An important challenge in this respect was that MDA targets the same epsilon amino groups on lysines that are also used to couple NHS dyes, and other coupling alternatives such as maleimide dyes targeting cysteines were not suitable in this case (data not included).

**Fig. 1:**
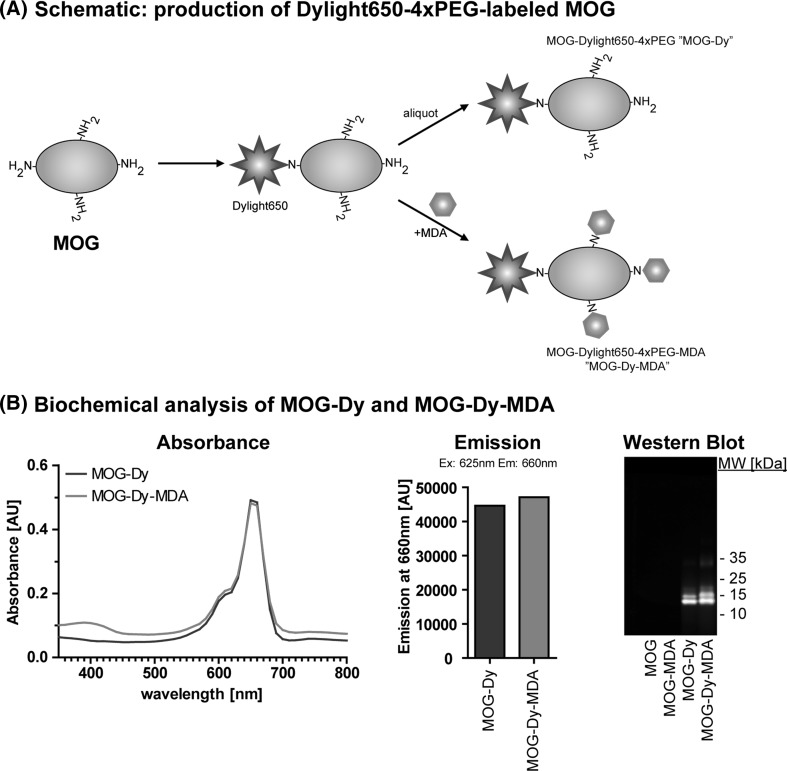
Fluorescently labeled and MDA-modified MOG to study uptake. **a** Schematic: production of Dylight650-4xPEG-labeled MOG. This schematic depicts the production of fluorescently labeled MOG or MOG-MDA. In an initial step, MOG is coupled with the fluorescent Dylight650-4xPEG dye. This step was optimized to yield a 1:1 molar ratio, modifying on average only one of the available lysines, leaving the remaining ones available for subsequent MDA modification using aliquots from the same batch. Notably, the PEGylated dye should not allow for any potential modification of the dye by MDA. **b** *Left* The absorbance spectra for equal protein inputs (25 µg/mL) analyzed by photometry. The spectra of either MOG-Dy (*blue*) or MOG-Dy-MDA (*red*) overlap apart from a peak at 400 nm characteristic of fluorescent MDA-derived adducts. Importantly, the excitation peak for the Dylight650-4xPEG dye is identical. Unmodified protein did not display any absorbance peaks in this range (not included). *Center* The emission at 660 nm of diluted protein aliquots (2.5 µg/mL) exited at 625 nm was analyzed using fluorescence spectroscopy. MDA modification did not interfere with the emission of the Dylight650-4xPEG dye. Protein without fluorescent label did not display emission at 660 nm (not included). *Right* Using the fluorescent properties of the protein, we scanned antibody-free Western Blot membranes using the Odyssey^®^ CLx Infrared Imaging System (700 nm *green* and 800 nm *red*). The fluorescent dye appeared in both channels yielding a *yellow* merge, but detection was restricted to labeled protein with equal signal. Dy = Dylight650-4xPEG (Color figure online)

As depicted in Fig. 1a, we optimized the reaction conditions to couple Dylight650-4xPEG to MOG in a 1:1 molar ratio, leaving 4/5 of the lysines in MOG unmodified. An aliquot of the labeled MOG (‘MOG-Dy’) was further modified using MDA, yielding fluorescently labeled MDA-modified MOG (‘MOG-Dy-MDA’). Using this procedure, we feared that MDA modification might affect the fluorescent dye through either the generation of fluorescent adducts or modification of the dye itself, the latter being the major motivation for choosing 4xPEGylated Dylight650 over non-modified dye. The final products underwent a thorough quality control: fluorescent MDA adducts typically increased absorbance at ~390 nm, but importantly there was no change in the absorbance peaks for the Dylight650-4xPEG at 650 nm and the product was stable (Fig. 1b). Secondly, the emission of MOG-Dy-MDA at 660 nm after excitation at 625 nm was identical to that of MOG-Dy when tested by fluorescence spectrometry (Fig. 1b). This would be expected if the fluorescent label was not affected by subsequent modification, given that MOG-Dy-MDA was always derived from the same batch of MOG-Dy. Furthermore, the emission range of the fluorescent MDA adducts in flow cytometry was around 450–550 nm and corresponds to primarily the FL9 (450/50 nm) and FL10 (550/40 nm) channels, while also bleeding into the FL1 channel (525/40 nm). The 650-nm dye (FL6 channel, 660/20 nm) was therefore chosen as this was not affected by fluorescent MDA adducts. Lastly, we used the fluorescent properties to scan antibody-free Western Blots using a Li-COR Odyssey^®^ CLx system, allowing us to assess the emission at the 700- and 800-nm channels using an alternative method. Again, there was no apparent difference in the signals (Fig. 1b).

Taken together these analyses ascertained that we succeeded in producing fluorescently labeled MDA-modified MOG that we could then use to study uptake.

### The Uptake of MDA-Modified Antigen is Significantly Enhanced and Mediated by Scavenger Receptor A

**Fig. 2:**
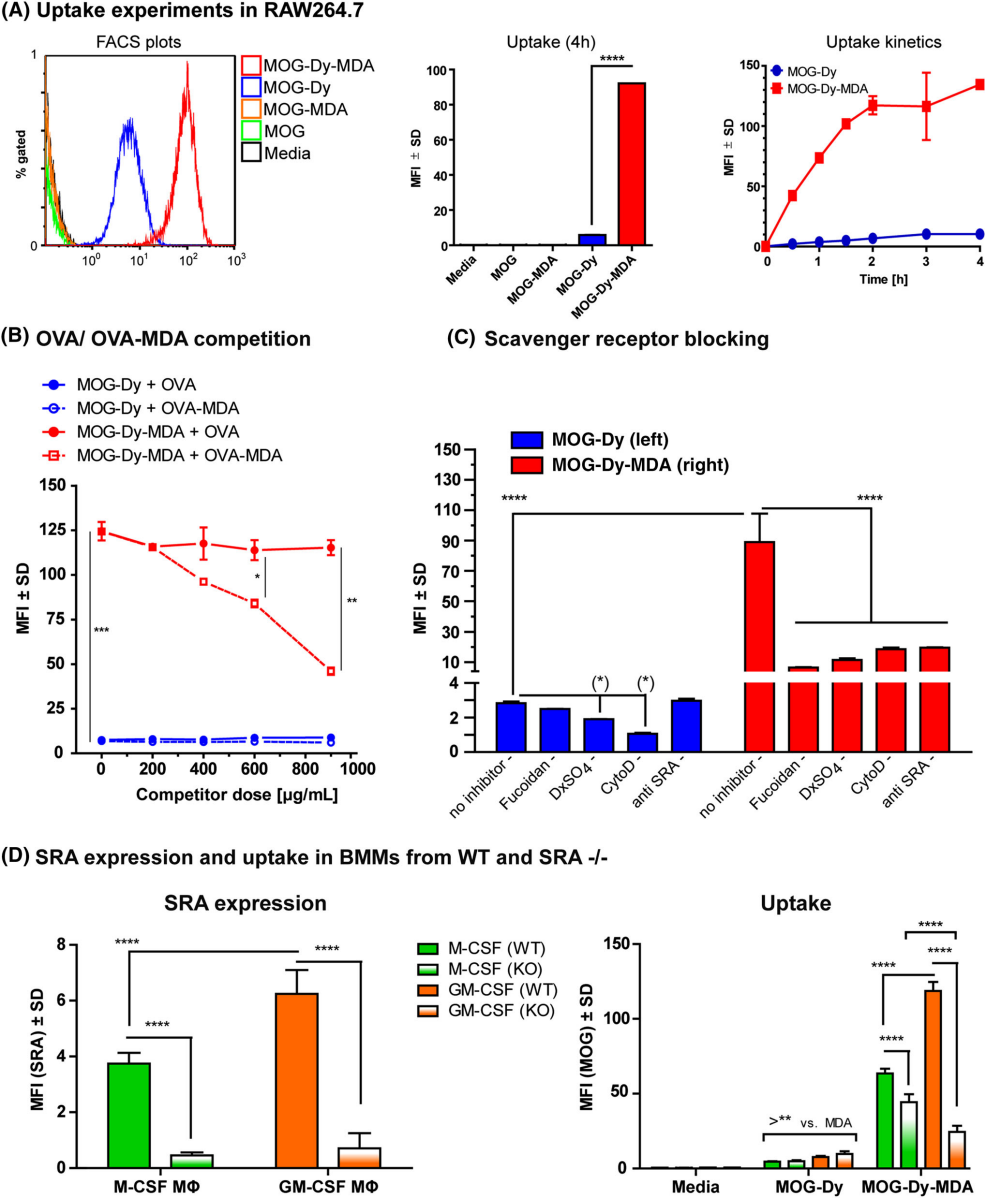
Enhanced uptake of MDA-modified MOG is SRA dependent and associated with M2 macrophages. **a** RAW264.7 macrophages were incubated up to 4 h with 10 µg/mL of protein and analyzed by flow cytometry. *Left* Histogram plots (distribution) over fluorescence intensity. Unlabeled proteins were negative, whereas labeled proteins yielded district peaks (MOG-Dy: *blue, center peak*, MOG-Dy-MDA: *red, rightmost peak*). *Center* The median fluorescence intensity (MFI) quantification of the replicated shows a ~tenfold higher uptake of MDA-modified MOG (*p* < 10^−4^, Student’s T Test). *Right* uptake kinetics for MOG-Dy and MOG-Dy-MDA (*upper curve*) indicating saturation at the 4-h timepoint. **b** Increasing doses of ovalbumin (OVA) or OVA-MDA were applied to compete with the uptake of MOG-Dy or MOG-Dy-MDA in RAW264.7 cells. OVA-MDA, but not OVA, was able to compete with the uptake of MOG-Dy-MDA, implying the involvement of a MDA-dependent receptor. **c** The uptake of MOG-Dy or MOG-Dy-MDA by RAW264.7 cells was performed in the presence of a panel of inhibitors or antibodies. DxSO_4_ = Dextran Sulfate; CytoD = Cytochalasin D; Cf. Supplementary Fig. 3 for a full panel. **d** Non-polarized primary bone marrow-derived macrophages grown with either M-CSF (*green*) or GM-CSF (*orange*) for WT B6 (*solid*) or SRA KO mice (gradient) were assessed for the expression of SRA (*left*) and uptake (*right*). Note that SRA signal in KO mice was background. Uptake of MOG-MDA was impaired in the SRA knockouts. Dy = Dylight650-4xPEG. *Asterisk* indicate *p* values as described (Color figure online)

As monocytes are one of the major pathogenic cells during neuroinflammation, we used the monocyte cell line RAW264.7 cells to study the uptake and fluorescent properties of unlabeled and labeled proteins, as well as uptake kinetics (Fig. 2a). Notably, the fluorescent signal was very bright, despite the comparably low doses of antigen applied (10 µg/mL). As expected, only fluorescently labeled MOG yielded significant signals by flow cytometry (Fig. 1c, left). Interestingly the uptake of MOG-Dy-MDA was significantly faster and roughly tenfold higher than that of MOG-Dy, and saturation was reached after 3–4 h (Fig. 2a).

We next investigated the mechanism of increased uptake of MDA-modified antigens. To establish that this was indeed an MDA-specific effect, we performed uptake assays in which we pre-incubated RAW264.7 cells for 30 min with unlabeled ovalbumin (OVA) or MDA-modified ovalbumin (OVA-MDA) before adding either MOG-Dy or MOG-Dy-MDA. In this setup, only increasing doses of OVA-MDA, but not OVA, were able to compete with the uptake of MOG-Dy-MDA (Fig. 2b). The doses to achieve this inhibition were comparatively high, indicating that these cells have a tremendous capacity to take up MDA-modified antigen. Despite this, the operational doses of Dylight650-4xPEG-labeled protein were kept low because the signals were already very bright.

These experiments thus established that there must be specific recognition of MDA by cellular receptors involved in the increased uptake of MDA-modified antigen. In order to explore the specificity of the uptake, we thus next performed uptake assays with RAW264.7 cells in the presence of a panel of scavenger receptor competitors, small molecule inhibitors and specific antibodies targeting various scavenger receptors or uptake mechanisms (Fig. 2c and Supplementary Fig. 3). From these data, it was apparent that the increased uptake of MOG-Dy-MDA could be inhibited by competitors for scavenger receptor A (Fucoidan and Dextran Sulfate) and specific blocking antibodies toward scavenger receptor A to a similar degree, as well as by a small molecule inhibitor of actin polymerization (Cytochalasin D). The latter even inhibited the uptake of MOG-Dy to some degree, as expected. Other tested targets, namely Mannose Receptor (CD206, inhibited by Mannan), SR-B1 (inhibited by BLT-1 or anti-SR-B1), CD36 (inhibited by anti-CD36) or MARCO (SCARA2, inhibited by anti-MARCO), did not inhibit MOG-Dy-MDA uptake.

Taken together these data highlight scavenger receptor A as the main mediator of elevated uptake of MDA-MOG.

For final confirmation, we tested bone marrow-derived macrophages from either wild-type or SRA knockout C57BL/6 mice. These macrophages were differentiated in media containing either M-CSF or GM-CSF, respectively (Mia et al. 2014) and subsequently used to measure uptake and expression of SRA by flow cytometry. Firstly, the degree of uptake correlated with the expression of SRA in wild-type macrophages (Fig. 2d). Secondly, the increased uptake of MOG-Dy-MDA was significantly reduced in macrophages from the SRA knockout strain (Fig. 2d), although not to the levels of MOG-Dy, and was similar to when using inhibitors (cf. Fig. 2c). This potentially implies the minor involvement or another receptor with overlapping ligand spectrum. In this context, it was previously demonstrated the MDA-modified low-density lipoprotein (LDL-MDA) is bound by CD36, LOX1 and FcγRIII, although at significantly lower magnitude compared to SRA (Zhu et al. 2014). Notably, there were apparent differences in expression of SRA and uptake depending on the macrophage differentiation state. In this case, GM-CSF-differentiated macrophages expressed higher levels of SRA and also exhibited higher uptake compared to M-CSF-differentiated macrophages.

In summary, these results confirm that scavenger receptor A is the main mediator of enhanced uptake of MDA-modified antigen, with quantitative differences depending on cell status.

### Expression of Scavenger Receptor A and Uptake of MDA-Modified Antigen is Associated with Anti-Inflammatory M2 Macrophages

**Fig. 3:**
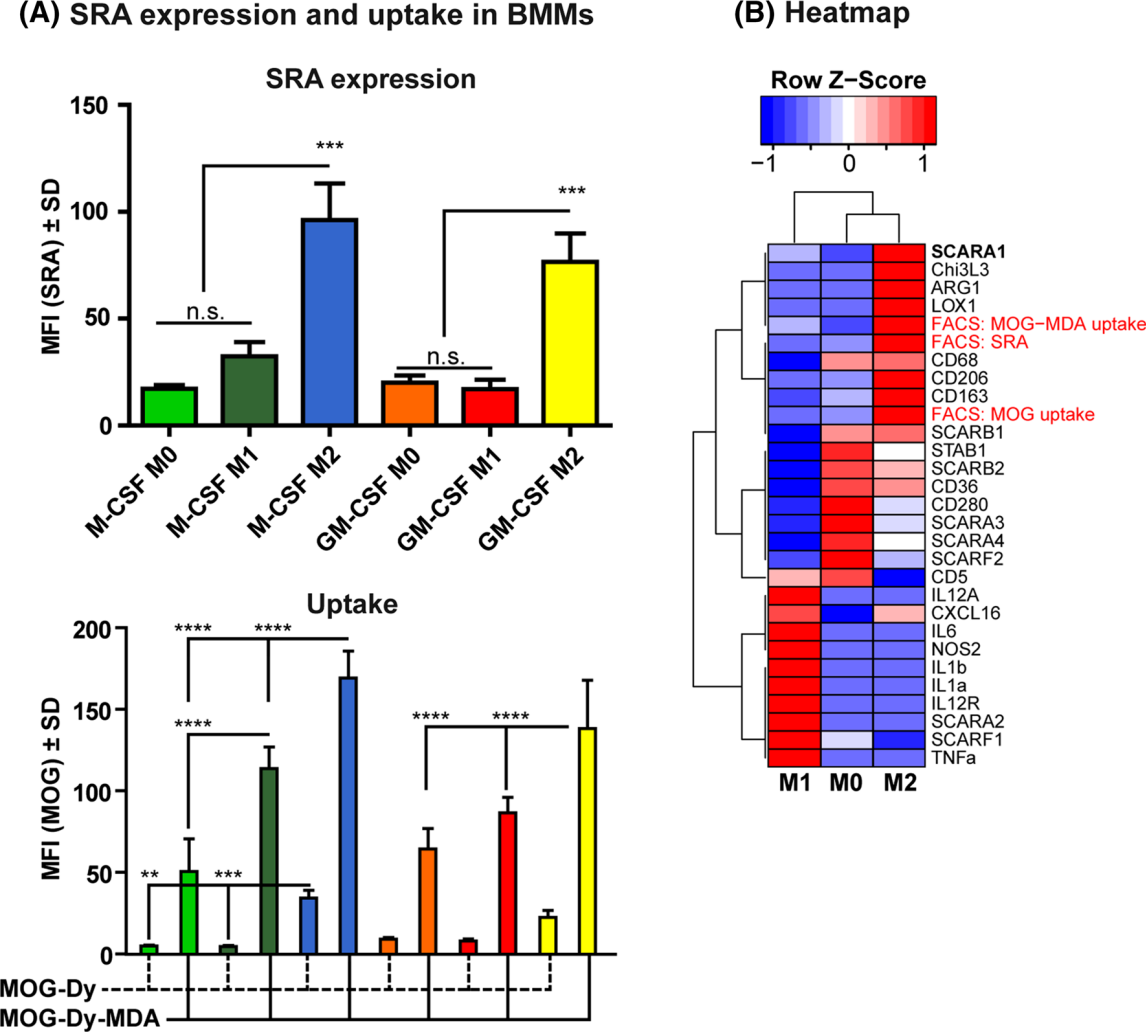
Expression of SRA and enhanced uptake are associated with M2 macrophages. **a** Primary bone marrow-derived macrophages from WT B6 mice were differentiated with either M-CSF or GM-CSF and polarized into M0, M1 or M2 states, respectively (cf. center legend). Flow cytometry was applied to assess SRA expression (*left*) and protein uptake (*right*). Dy = Dylight650-4xPEG. Stars indicate p values as described. **b** Polarized macrophages were analyzed for their expression of polarization markers and scavenger receptors by RT-PCR. The RT-PCR data were combined with FACS data for SRA expression and uptake (indicated in *red*). A heatmap using unsupervised clustering was generated using the replicates’ z-scores for each category. High uptake clusters together with SRA expression (both by PCR and FACS) and markers for the M2 phenotype (e.g., ARG1, Chi3L3, CD206). Though the highest expression of scavenger receptors differs for the respective polarizations, generally the expression of scavengers clusters together with M2 or M0 phenotypes (Color figure online)

We followed up on the notion that the phenotype of the macrophage had influence on the expression of SRA and uptake of MDA-modified antigen. We postulated that the uptake would also differ depending on the activation state of macrophages, again correlating with the expression of SRA. To explore this, we assayed macrophages for uptake after having polarized them to conditions that resemble two extremes of pro- or anti-inflammatory phenotypes induced by LPS/IFNγ (M1) or IL-4/IL-10/TGFβ (M2) polarizations, respectively (Mia et al. 2014). Strikingly, the expression of SRA was significantly induced in the M2 phenotypes (Fig. 3a, top) and the uptake of MOG-Dy-MDA in M2 polarized macrophages surpassed that of M1 polarized macrophages (Fig. 3a, bottom). The baseline uptake of MOG-Dy was elevated in M2 macrophages, furthermore indicative of their generally higher phagocytic capacity.

To support these data, we also analyzed polarized macrophages using RT-PCR for a panel of polarization markers and several scavenger receptors (Canton et al. 2013; Mia et al. 2014) and combined this with FACS data for SRA expression and uptake. From this collective data, we generated a heatmap using unsupervised clustering according to the respective polarization types and the z-scores for the respective categories. Uptake of MDA-modified antigen clustered together with the expression of SRA (FACS) or its gene *SCARA1*, along with markers of the M2 phenotype, namely *Chi3L3* and *ARG1*, as well as other scavenger receptors: *LOX1*(Zhu et al. 2014), *CD68*, *CD206* and *CD163* (Fig. 3b). Although this does not establish causality, this clustering clearly underlines the correlation between SRA expression and uptake of MDA-modified antigen. Importantly, we had already established functional causality for SRA mediating the uptake of MDA-modified antigen (cf. Fig. 2).

In conclusion, these data establish a clear association of SRA expression and uptake of MDA-modified antigen with M2 macrophages.

### MDA Modification Can Protect MOG from Proteolytic Digestion In Vitro, but Intracellularly MOG-MDA is Targeted for Digestion

**Fig. 4:**
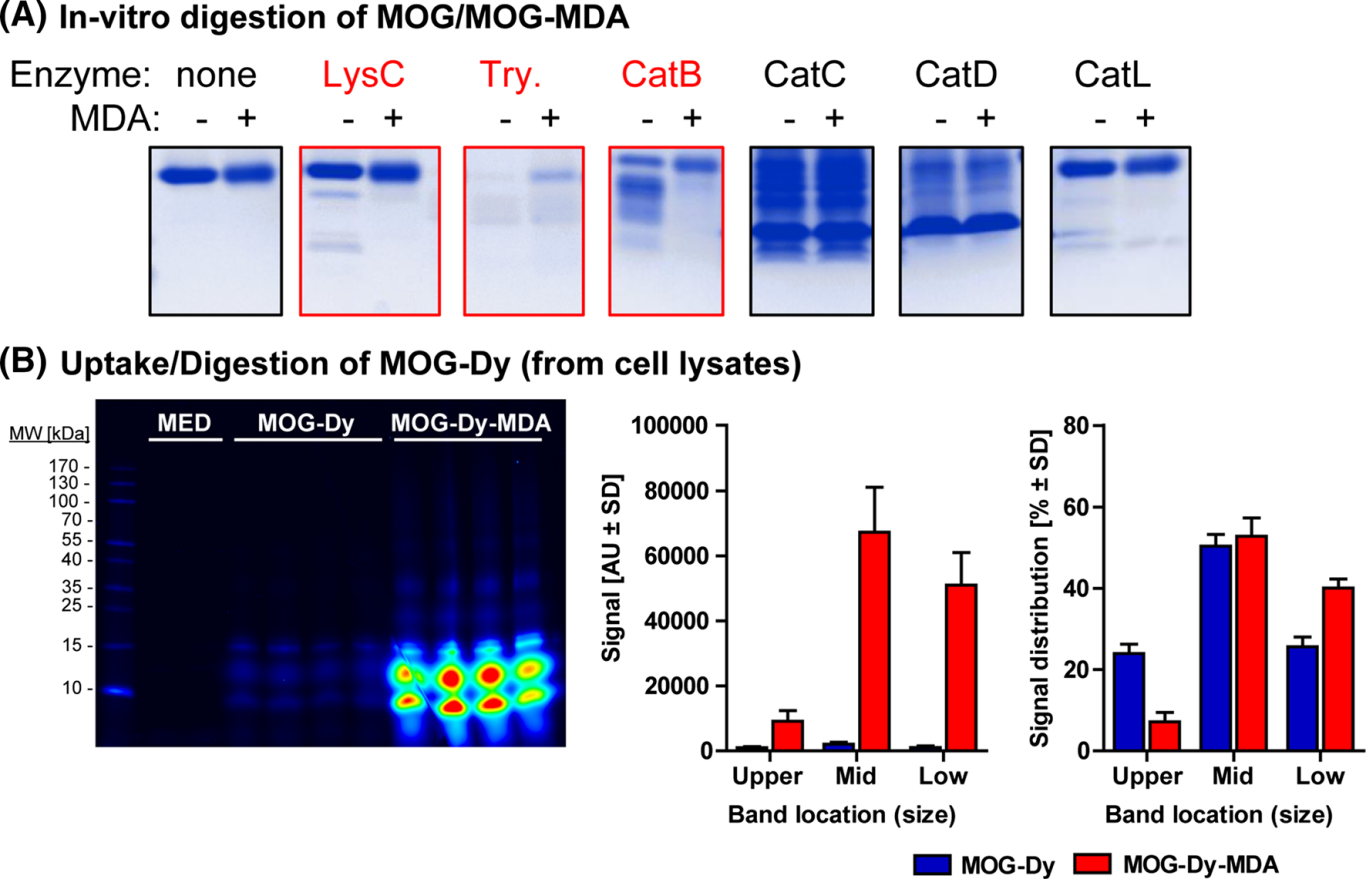
MDA modification denies digestion of the carrier antigen by lysine-dependent proteases, but MDA-modified antigen is digested upon uptake into lysosomes. **a** MOG (−) or MOG-MDA (+) were digested in vitro with the indicated enzymes, separated by Tricine gel SDS-PAGE and stained with Coomassie* Blue*. MOG-MDA was protected from proteolytic digestion by the highlighted enzymes. *Try* Trypsin; *Cat* Cathepsin. **b** RAW 264.7 cells were incubated with 10 µg/mL MOG-Dy or MOG-Dy-MDA for 4 h, then washed twice and processed to obtain protein lysates. Equal aliquots were separated by Tricine gel SDS-PAGE, and the gels were scanned on the Odyssey^®^ CLx Infrared Imaging System (700 nm channel, intensity scaling). The signals were quantified using the software. *Left* representative image of a scanned gel with indicated sample replicates. *Right* quantification of the raw signal and relative signal distribution (Color figure online)

An important question was to address the fate of the phagocytosed antigen, and whether MDA modification would interfere with the proteolytic digestion of MOG. First we digested MOG or MOG-MDA in vitro using a panel of commercially available purified enzymes and separated the digests using Tricine gel electrophoresis. These analyses revealed that MDA-MOG was protected from digestion by Lys-C, Trypsin and Cathepsin B (Fig. 4a). Lys-C cuts specifically after lysines while Trypsin cuts after lysines or arginines, respectively. CatB is proposed to have a more promiscuous motif (Biniossek et al. 2011). Our results thus strongly imply that MDA modification denies the recognition and digestion by proteases that depend on lysine since these residues are modified by MDA. The lysine-specific Lys-C is a bacterial enzyme and serves as a proof-of-concept in this setting. In contrast, Trypsin and especially the lysosomal Cathepsins are more physiologically relevant candidates, although the proteolytic enzymes that would digest MOG in vivo remain to be identified. Importantly, other enzymes, namely CatC, CatD or CatL, digested MOG regardless of modification.

To assess the digestion in cells, we again exploited fluorescently labeled MOG-Dy and MOG-Dy-MDA. Since these variants were randomly tagged with the dye, we could expect the dye to be present in several fragments generated by intracellular digestion. We thus incubated RAW264.7 cells with the labeled protein and separated the obtained lysates using Tricine gel electrophoresis, allowing us to scan the gel and visualize the fragments. This analysis revealed two primary observations: First, the bands for MOG-Dy-MDA were much more intense than were those for MOG-Dy (Fig. 4b), confirming the uptake data obtained by FACS. Secondly, a more detailed analysis of the relative signal distributions between the original MOG band (~15 kDa) and lower fragments (~12.5 or ~10 kDa) revealed that the lowest band was more intense in MOG-Dy-MDA at the expense of the original band. Together these data suggest that MOG and MOG-MDA are digested in lysosomes upon uptake. Furthermore, they suggest that MOG-MDA is digested faster than is MOG, because the relative signal for fragments was more intense (Fig. 4b) and the fact that MOG-MDA is harder to detect in cell lysates using Western blotting (cf. Suppl. Fig. 2B). However, these observations may simply reflect the fact that MOG-MDA is taken up at a higher rate (cf. Fig. 2a) and thus has a head start with regard to digestion.

In conclusion, although MDA modification clearly protects MOG from digestion by lysine-dependent proteases, this does not appear to affect the fragments yielded by digestion in cells, although rates may differ. Accordingly, the rate of uptake is likely to accelerate the delivery to lysosomes and the start of digestion.

### MDA-MOG Maximized the Proliferation of MOG-Reactive 2D2 Splenocytes In Vitro, but Does not Induce More Severe EAE in Wild-Type C57BL/6 Mice In Vivo

**Fig. 5:**
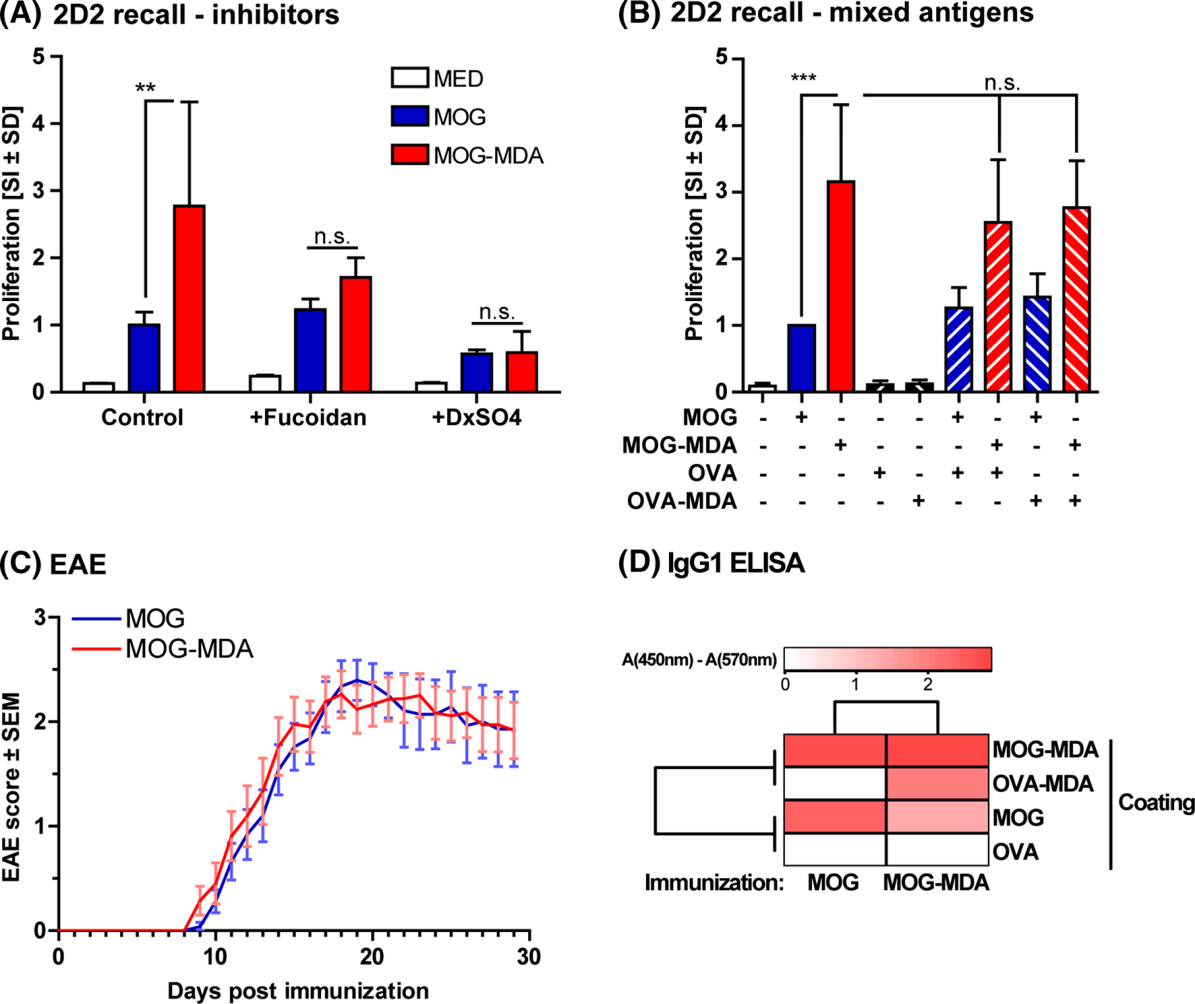
Enhanced uptake of MDA-modified MOG maximized the proliferative potential of TCR-transgenic 2D2 cells, but does not increase encephalitogenicity. **a** Splenocytes from MOG_35-55_-specific TCR transgenic 2D2 mice were used to perform Tymidine incorporation assays in the presence of 50 µg/mL and respective SRA inhibitors. *SI* stimulation index (relative to MOG). The antigen-specific proliferation is maximized for MOG-MDA, an effect that is prevented by SRA inhibitors. **b** Proliferation assays were performed as above, but in the presence of the respective protein mixtures as indicated. The increased proliferation requires MDA to be attached to MOG in order to elicit the effect. **c** WT C57BL/6 mice immunized with either MOG or MOG-MDA to evoke the EAE model as described. The paralysis score was assessed daily. Data are a meta-analysis of 4 independent experimental repeats (*n* up to 25/group total). The groups displayed no change in EAE course or severity. **d** Sera from EAE animals were obtained at the experimental endpoint and assayed in an IgG1 ELISA assay for the indicated coating antigens. The absorbance values are displayed as a heatmap, redness correlating to higher absorbance (cf. scale). Note the specific presence of antibodies against OVA-MDA; but not OVA for mice immunized with MOG-MDA. *OVA* ovalbumin (Color figure online)

Up to this point, the results had established that MOG-MDA was taken up at a higher rate via scavenger receptor A and was digested in cells. To put these observations into a biological context, we addressed whether MDA modification would affect the antigenicity of the carrier protein. First we made use of TCR transgenic 2D2 mice that have sole T cell reactivity to MOG_35-55_. Proliferation assays established that in this system MOG_35-55_-reactive T cells proliferated roughly threefold more toward MOG-MDA than to MOG (Fig. 5a). The enhanced proliferation can be attributed to optimized uptake, since inhibition using Fucoidan or Dextran Sulfate diminished this effect (Fig. 5a). Secondly, we established that the increased proliferation required the MDA modification to be on the target antigen, as co-incubation of MOG with OVA-MDA did not induce bystander activation, 2D2 splenocytes only proliferating more strongly toward MOG-MDA (Fig. 5b). Notably, competition of uptake using doses of OVA-MDA at 50 µg/mL was negligible in this setup. Together these results indicate that maximized uptake of MOG-MDA via SRA can enhance the proliferation of MOG-specific T cells in vitro.

With MDA modification being able to enhance the proliferation of autoreactive T cells in vitro, we next addressed whether MOG-MDA would elicit more severe experimental autoimmune encephalomyelitis (EAE) in vivo in wild-type C57BL/6 mice. Interestingly, these experiments did not indicate any difference in clinical EAE severity (Fig. 5c). We recovered serum samples from these mice at the experimental endpoints and performed custom ELISAs to assess antibody titers toward MOG, MOG-MDA, OVA or OVA-MDA, respectively. These analyses revealed that there were clear titers of IgG1 toward MOG and MOG-MDA in both groups (Fig. 5d), the titers toward MOG-MDA being higher in mice immunized with MOG-MDA. However, there was also significant reactivity toward OVA-MDA, but not OVA, in MOG-MDA immunized mice. This antibody reactivity to MDA indicates that immunization with MOG-MDA induces responses not only to MOG, but also to the MDA adducts.

Taken together these results are somewhat surprising. In vitro, MDA-MOG clearly has the capacity to enhance the proliferation of MOG-reactive 2D2 T cells, but this difference does not manifest in more severe EAE in immunized wild-type mice in vivo. Furthermore, the antibody response induced by MOG-MDA included reactivities to both MOG and the MDA adduct itself. These results have important implications that we discuss below.

## Discussion

### Results Summary

Herein we have made use of fluorescent labeling to investigate the uptake and fate of MDA-modified MOG in macrophages. MDA-modified MOG is taken up significantly more effectively via scavenger receptor A; an effect that is most strongly associated with homeostatic M2-type macrophages. The phagocytosed MOG is digested indifferently and enhances the proliferation of MOG-reactive 2D2 splenocytes in vitro. However, immunization of wild-type C57BL/6 mice with MOG-MDA does not induce a more severe EAE phenotype in vivo, and the induced antibody response includes reactivity toward both MOG and the MDA adducts.

### The Uptake of MDA-Modified Antigen Mediated by SRA is Highly Effective and Associated with M2 Macrophages

Using fluorescently labeled MOG and the MDA-modified equivalent, we have demonstrated that MDA-adducted MOG is taken up via scavenger receptor A, and the importance of SRA in mediating uptake of antigen-bearing MDA or related maleyl modifications has already been described (Weismann and Binder 2012). Reports include both binding to SRA (Willis et al. 2004; Berger et al. 2014; Shechter et al. 1981) and SRA-dependent immune responses to modified antigen (Nicoletti et al. 1999; Willis et al. 2003). Although in previous cases the binding to SRA was mostly implicated by inhibition of proliferation assays using Fucoidan, we herein extend these findings and directly demonstrate SRA dependence using competitors, small molecules, specific blocking antibodies and SRA knockout mice. Furthermore, the use of thoroughly controlled custom fluorescently labeled and MDA-modified probes enabled us to quantify the relative differences in uptake, which was in the range of ~tenfold more effective when mediated by SRA. Furthermore, the effectiveness increased with higher SRA expression, as was demonstrated to be the case for M2 macrophages. Although the exact value may differ depending on cell type, SRA expression, antigen and dose, the effect is very clear: the uptake of MDA-modified antigen is significantly more efficient compared to that of native antigen.

A novel observation in this work is the association of SRA-dependent uptake of MDA-modified antigen with M2 macrophages, the expression of SRA in particular, and scavenger receptors in general being highly elevated in alternatively activated M2-type macrophages (Canton et al. 2013; Mia et al. 2014; Mantovani et al. 2013; Kou and Babensee 2011; Wynn et al. 2013). This feature highlights the capacity of M2 macrophages to contribute to phagocytic clearance of antigen and tissue remodeling and homeostasis. Notably, this observation has important implications for the role of MDA-modified antigen in vivo. Since M2 macrophages are not expected to initiate inflammation, but rather to promote its resolution, the presence of MDA-modified antigen is therefore primarily expected to constitute a sterile ‘eat me’ clearance signal above anything else. This agrees with reports demonstrating that MDA adducts constitute a ligand for complement factor H (Weismann et al. 2011) or natural antibodies of newborns (Wang et al. 2013; Binder 2010; Chou et al. 2009), among other oxidation-specific epitopes. Secondly, SRA has several anti-inflammatory roles (reviewed in Canton et al. 2013). In this context, an adapted concept is emerging, where MDA epitopes primarily constitute clearance signals, while non-physiological accumulation may promote sterile inflammation (Busch and Binder 2017).

However, the proposal that MDA is primarily a clearance signal is challenged by reports that would suggest otherwise. Among others, MDA adducts have been suggested to: increase the antigenicity of its carrier protein (Wallberg et al. 2007; Wuttge et al. 1999); enhance T cell proliferation (Willis et al. 2002); be recognized by complement factor C3a (Veneskoski et al. 2011); entail homeostatic problems in cells (Höhn et al. 2014; Willis et al. 2004); potentially alter signaling (Ott et al. 2014); constitute a co-factor for LPS (Duryee et al. 2004); and even be immunogenic per se in the absence of adjuvant (Thiele et al. 1998). Furthermore, other studies suggest roles for anti-MDA antibodies in atherosclerosis (Antoniak et al. 2015; Duryee et al. 2010) and cardiovascular mortality (Maiolino et al. 2013).

In light of these apparently contradicting reports, it is difficult to discern an apparent consensus. Importantly, the pro-inflammatory functions of MDA adducts appear mostly to be manifest at high doses, corresponding to those that also induce cell death (Willis et al. 2002). Hence, the pro-inflammatory effects attributed to MDA adducts may instead be attributed to traces of soluble MDA remaining in the protein preparation due to insufficient dialysis, or possibly relate to endotoxins in the starting material being cross-linked by MDA to the carrier protein. Regarding the first point, we have evidence from pilot experiments using MDA-modified MOG in 2D2 T cell proliferation assays in which insufficiently dialyzed protein-induced peak proliferation before turning toxic with progressive doses (data not included). Importantly, cell death triggered in vivo at high doses may induce bystander activation due to the release of DAMPs from dying cells which may account for the pro-inflammatory activation (Thiele et al. 1998). In this context, it is important to bear in mind that the preparation of MDA-modified antigen in vitro is not necessarily representative of the modification or doses in vivo (Gutteridge 1975; Millanta et al. 2013). Great caution is advised for the preparation of MDA modifications as otherwise the effect of soluble component or adduct cannot be reliably dissociated.

In conclusion, we postulate that MDA adducts primarily constitute a clearance signal for M2-type macrophages. Importantly, however, this does not necessarily exclude ensuing pro-inflammatory activities should the clearance capacity be overwhelmed by extensive cell death or accumulation of scavenger ligands. This implies that the homeostatic clearance system for MDA adducts, consisting of SRA, natural antibodies (Wang et al. 2013; Binder 2010; Chou et al. 2009) and complement factor H (Weismann et al. 2011), may have a limiting buffer before inflammation is induced, involving, e.g., C3a (Veneskoski et al. 2011) and tissue-infiltrating monocytes. In this context, the balance between pro- or anti-inflammatory cells and stimuli may determine the final immunological outcome. The recognition of MDA adducts by complement C3 has been demonstrated using human serum (supplementary data of Veneskoski et al. 2011), but could not be detected using purified C3 in a previous study (Weismann et al. 2011), and the binding of C3 to MDA adducts could therefore be indirect via factor H. If so this would argue in favor of an anti-inflammatory role of innate recognition of MDA adducts. Furthermore, it is recognized that SRA may have differing roles depending on the involvement of co-receptors that can influence the cellular signaling and response (Canton et al. 2013).

### MDA Modification Can Affect Antigen Digestion at Several Levels

Herein we have demonstrated that MDA adducts negatively affect the digestion of MOG by lysine-dependent proteases such as LysC (lysine-specific) or Trypsin (lysine or arginine), as well as Cathepsin B. A straightforward explanation for this observation is that MDA targets epsilon amines of lysines and the fact that MDA-modified protein has a lower isoelectric point, i.e., a more negative net charge. Both the physical obstruction and lack of positive charge could thus prevent the digestion of MDA-modified antigen by lysine-dependent proteases. Interestingly, compared to LysC or Trypsin, Cathepsin B has been proposed to have a more promiscuous restriction motif, not involving a lysine (Biniossek et al. 2011). However, the method applied to identify this motif in that study involves a biotinylation of lysines, a factor that may physically interfere with digestion of motifs harboring a biotinylated lysine.

In an earlier study addressing the proteosomal stability of MDA-modified antigen, it was demonstrated that the presence of MDA-modified antigen itself interferes with the co-digestion of unmodified antigen (Kaemmerer et al. 2007). This implies that the inhibition of proteolytic cleavage also potentially applies to other proteins than the modified antigen itself. When we probed the digestion pattern of fluorescently labeled MOG in cell lysates, there was no indication that the overall pattern differed (Fig. 4), although there seemed to be changes in relative abundance. Possibly the head start in uptake and effective feeding toward the cells’ proteolytic machinery may accelerate the initial rate of digestion. However, identifying the processing enzymes involved in various APCs and resulting peptides requires separate detailed study.

The fact that MDA-modified antigen potentially has resistance to proteolytic cleavage has immunological implications: (1) the antigen itself may have an extended half-life in the extracellular space due to its resistance to proteolysis; (2) depending on the antigen the proteolytic cascade may be entirely altered, as it was previously demonstrated that a single processing site may determine the ensuing processing events (Antoniou et al. 2000); (3) proteolytic cleavage may dictate whether a potential immunodominant epitope is either destroyed by ‘destructive processing’ (Manoury et al. 2002), or otherwise a cryptic epitope can be released as a consequence of non-canonical processing (Stoeckle and Tolosa 2010; Doyle et al. 2007); 4) the bulky MDA adduct may physically interfere with the presentation of a modified epitope by obstructing MHC-anchoring pockets or representing an excessive TCR-facing residue. Lastly, the overwhelming accumulation of damaged, modified and aggregated antigen in macrophages may result in a failure to maintain homeostasis (Höhn et al. 2014) or defective lysosomal integrity at excessive doses (Willis et al. 2004). For the latter scenario, we observed no evidence for MDA-adducted protein-induced toxicity at the doses we used (50 µg/mL vs. 500 µg/mL in (Willis et al. 2004)), but there is evidence that cell death and lysosomal instability can be induced by the MDA-derived chemicals (data not included).

The discussed data and implications delineate how pivotal processing events are for the generation, presentation and recognition of epitopes from a given antigen (Stoeckle and Tolosa 2010), and how a modification such as MDA adducts may interfere with this cascade. However, in the case of MDA-modified MOG our data did not support any obvious alterations of its digestion in macrophages, even though the digestion in vitro was clearly affected for LysC, Trypsin and Cathepsin B. The enzymes that digest MOG in different APCs, however, remain to be identified and possibly differ depending on polarization or activation state.

### Implications for the Adaptive Immune Responses Toward MDA-Modified Antigen

We studied the role of MDA adducts in adaptive immunity using the CNS-specific antigen MOG. Using the MOG_35-55_ reactive TCR transgenic 2D2 mice (C57BL/6 background), we observed a significant increase in proliferation in vitro using MOG-MDA compared to native MOG and demonstrated that this was in fact attributable to degree of uptake. This in vitro observation stands in contrast to what we observed in vivo. The EAE model did not reveal significant differences between either MOG or MOG-MDA as antigen, and re-stimulation of lymph nodes yielded similar proliferation (Suppl. Fig. 4).

We can explain this discrepancy firstly by the fact that the 2D2 system has a rich abundance of antigen-specific T cells that compete for presented antigen, which is the limiting factor for proliferation. Hence, with maximized uptake the proliferation is maximized accordingly. In re-stimulation assays of ex vivo lymph nodes, however, the abundance of antigen-specific T cells is several orders of magnitudes lower, and the few antigen-specific T cells that are present do not face competition. Thus, in this case the availability of antigen is not a limiting factor, unless one would first grow T cell clones for re-stimulation or limit the antigen in a dilution series. This also raises questions about potential competition between M2-type cells and other APCs in the 2D2 in vitro system. That the EAE experiments did not display any pathological difference implies that MDA-modified MOG is equally encephalitogenic compared to native MOG. Importantly, the C57BL/6 EAE model is heavily skewed to induce autoimmune disease even with the native MOG and involves the use of complete Freund’s adjuvant and pertussis toxin in order to evoke CNS inflammation. In light of this strong stimulation, it is difficult if not impossible to detect differences that may pertain to the immunogen itself.

We have demonstrated that the uptake of MDA-modified MOG is mediated by SRA. However, SRA expression is almost exclusive to macrophages and the monocytic lineage, whereas classical dendritic cells, specifically those differentiated under FLT3L as opposed to GM-CSF monocyte-derived cells (Guilliams et al. 2014; N’diaye 2016), do not express SRA. Thus, it is questionable whether classical DCs that prime naïve T cells even have the capacity to take up MDA-modified antigen at a higher rate and promote licensing of T cells to begin with.

A previous study using DBA1 mice immunized with MDA-modified rat MOG reported it to be more encephalitogenic (Wallberg et al. 2007). Using mouse MOG, we could not reproduce this observation, even in DBA1 mice (data not included). Furthermore, in contrast to mouse MOG, rat MOG becomes highly insoluble after MDA modification and precipitates heavily. Moreover, the DBA1 epitope, MOG_79-96_ (GKVTLRIQNVRFSDEGGY) (Abdul-Majid et al. 2000) contains an amino acid substitution between rat (82 = *A*) and mouse (82 = *T*, underlined). A potential explanation lies within the observation that immunization of C57BL/6 mice with human MOG induces EAE through alternative mechanisms (Oliver et al. 2003), namely depending on B cell APC function (Molnarfi et al. 2013). This is attributed to a S42P substitution within the C57BL/6 MOG_35-55_ epitope between rodents and humans, and hence the dependence on initiating a secondary immune response to the murine epitope. If the corresponding scenario is the case in DBA1 mice immunized with MDA-modified rat MOG, a possible explanation could be that MDA-reactive B cells potentially contribute to initiating T cell responses toward MOG. However, whether this is actually the case or instead technical or other issues account for this discrepancy remains an open question, but the scenario is simultaneously considerably artificial. Importantly, we have demonstrated herein that immunization of C57BL/6 mice with MOG-MDA induces comparable EAE, but antibody responses toward both MOG and the MDA adducts. Accordingly, it would be highly interesting to study the role of modification-specific B cells in promoting autoimmunity toward carrier antigens.

### Interaction of Scavenger Receptors and Adaptive Immunity

Another previous study using SRA knockout mice in the MOG_35-55_ peptide-induced EAE model claimed that SRA is important for the induction of CD4^+^ T cell responses (Levy-Barazany and Frenkel 2012). In that study, MOG_35-55_-immunized SRA knockout mice displayed significantly milder EAE, less demyelination and lowered cytokine production (Levy-Barazany and Frenkel 2012). A recent study used fluorescently labeled antigen to demonstrate an SRA-dependent traffic of antigen from capturing B cells to macrophages (Harvey et al. 2008). Given that SRA knockout mice developed milder EAE (Levy-Barazany and Frenkel 2012), this may imply that the SRA-dependent transfer mechanism (Harvey et al. 2008) could be involved in sustaining the immune response in EAE. It is an open question whether this mechanism requires the antigen to be an SRA ligand or not. In our case, we controlled the fluorescent labeling to achieve a 1:1 molar ratio, avoiding the antigen being fully fluorescently labeled at all lysine residues because this may actually turn the antigen into an SRA ligand. In fact the uptake of fluorescently labeled MOG itself also weakly correlated with SRA expression, although not to the same degree as the MDA-modified version.

In regard to EAE, it appears that SRA-deficient mice already display shortcomings in the primary induction (Levy-Barazany and Frenkel 2012), although it cannot be excluded that SRA may play a role at later stages such as during demyelination by CNS-infiltrating monocytes. Furthermore, deficits in SRA-dependent structural organization of the marginal zone may influence the immunization process (Canton et al. 2013). Conversely, a recent study reports the opposite, namely that SRA-deficient APCs have a higher capacity to induce CD4^+^ T cell responses to ovalbumin (Yi et al. 2012). The authors attributed this to regulatory mechanisms of SRA (Yi et al. 2012), which would emphasize the notion of homeostatic clearance mechanisms mediated by SRA^+^ macrophages. In our study, there was no apparent difference in the immune response or EAE, despite MOG-MDA clearly being a SRA ligand, but it is unclear whether the strong adjuvant may mask minor effects in this model of autoimmune disease.

Importantly, the involvement of co-receptors of SRA may regulate (auto)immune responses, depending on whether PAMPs (e.g., via TLR4), or instead endogenous ‘eat me’ signals (e.g., via MERTK), are co-ligated (Canton et al. 2013). It is interesting that the respective signals counteract each other, i.e., MERTK ligation inhibits TLR signaling. The latter is especially relevant for the concept of ‘sterile inflammation’ in the absence of PAMPs. Conversely, SRA^−/−^ mice are hyper-sensitive to TLR-mediated systemic shock and production of TNF or IL-6, implying that co-ligation of SRA modulates the capacity of TLR signaling itself and that bacterial clearance is facilitated via SRA (reviewed in Platt et al. 2002). Accordingly, we report the association of phagocytosis of MDA-modified antigen with M2 macrophages via SRA and propose that MDA adducts primarily constitute clearance signals of stress-induced modified self-antigens via SRA. However, the response may potentially be fine-tuned depending on co-ligation of additional receptors. Taken together, however, the role of SRA in regulating immune responses is still largely elusive and more studies will be required to elucidate its contribution regarding specific cell populations and (modified) antigens.

## Conclusions

Here we have demonstrated that the uptake of MDA-modified antigen is mediated by scavenger receptor A and is most efficient by M2-type macrophages. The fact that the efficient uptake accounted for enhanced proliferation of specific 2D2 T cells in vitro is contrasted by the lack of difference in immunized wild-type mice in terms of immune priming or EAE disease course. Taken together, however, the results support the notion that MDA adducts primarily represent clearance signals for phagocytes. These would recognize and internalize the damaged and modified antigen via SRA, but subject it to immunological screening by previously licensed T cells involving antigen processing and presentation. In this context, the type and activation state of the APCs and their digestive capacity and surface receptor composition in relation to the composition and abundance of (modified) antigen all likely complexly contribute to shaping the immune responses during either homeostatic responses to isolated cell damage or later stages of inflammation.

## Electronic Supplementary Material

Below is the link to the electronic supplementary material.

Supplementary Figure 1 Biochemical analysis of MOG and its modified variants using the MDA or MAA protocols. Recombinant mouse Myelin Oligodendrocyte Glycoprotein was purified and modified with MDA, or MDA with addition of acetaldehyde (MAA), as described in the Methods. A) Analysis by Western Blotting: The proteins can be detected using anti HIS-tag antibodies. Some crosslinking by MDA occurs as evident by the dimeric band at ~35 kDa. Notably, the recognition of MOG-MDA or MOG-MAA by the anti-MOG monoclonal 8-18C5 antibody is reduced. This antibody is conformational, but also the modified protein may bind differently to the nitrocellulose membrane. Importantly, the recognition by MDA antiserum is stronger for MOG-MAA than for MOG-MDA, implying that the generation of the recognized epitope is enhanced, presumably the MAA adduct. Similar results were obtained using the 1F83 anti-MAA monoclonal (not included). B) Analysis by isoelectric focusing: Proteins were focused according to isoelectric point (pI) as described and stained with Coomassie Blue. The modified variants display a drop in pI, indicating an increase in net negative change (loss of positive charge). The pattern between MOG-MDA and MOG-MAA is similar. In this article we used MOG modified according to the MAA protocol with addition of acetaldehyde, but refer to it as MOG-MDA to respect the fact that the chemical reaction in vitro yields a spectrum of MDA-dependent adducts (TIFF 588 kb)

Supplementary Figure 2 Intracellular HIS-tag staining cannot be used to accurately study protein uptake. RAW264.7 cells or primary bone marrow-derived macrophages (BMMs) from C57BL/6 mice differentiated with either M-CSF or GM-CSF were cultures as described. The cells were incubated with recombinant (HIS-tagged) MOG or MOG-MDA (50 µg/mL or as indicated) for 4h. Cells were either prepared with intracellular FACS staining using or used to obtain lysates that were analyzed by Western Blotting using the indicated antibodies. A) FACS analysis: Left: RAW264.7, Right: primary bone marrow-derived macrophages. There was a profound background revealed for permeabilized cells, even in the absence of HIS-tagged MOG. The method failed to reliably detect the uptake of HIS-tagged MOG as the differences to media samples were mostly insignificant. Furthermore, there was no apparent effect with increased doses or MDA-modified MOG. B) Analysis by Western Blotting: The purified anti HIS-tag antibody was the same clone as the one used for FACS. Western Blotting revealed clear off-target staining of endogenous antigens (>70 kDa), both in RAW264.7 cells and in primary BMMs. The separation by SDS-PAGE allowed discrimination of bands corresponding to MOG at 15 kDa. The band for MOG-MDA appears to be weaker, which contradicts the observations from uptake experiments performed using the fluorescently labeled protein (cf. Figs. 1, 2). Importantly, the detection of unlabeled MOG uptake using Western Blotting is hampered by the immediate proteolytic digestion, as demonstrated in Fig. 4 of the main article. Taken together, these results establish that intracellular HIS-tag staining cannot be used to study uptake in these cells due to the background given by endogenous, intracellular targets and the inefficiency of the method to reliably detect phagocytosed antigen, along with proteolysis of phagocytosed antigen (TIFF 788 kb)

Supplementary Figure 3 Full panel of uptake inhibitors and blocking antibodies and isotype controls. This panel supports Fig. 2B of the main article, but includes samples that did not show any effect, i.e., Mannan, BLT1, anti-SRB1, anti-CD36, anti-MARCO (SCARA2), and various isotype controls. For further details we refer to the main article (TIFF 255 kb)

Supplementary Figure 4 FACS analysis of day 7 lymph nodes and proliferation. Day 7 lymph nodes (D7LN) from EAE-immunized B6 mice were collected and cells analyzed by flow cytometry, or incubated with 50 µg/mL of antigen for either thymidine incorporation assays or FACS analysis after recall, as described. A) APC panel: Lymphocytes were gated on singlet, live cells and then on CD11c^+^CD19^−^ (CD11c^+^ APCs) or CD11c^−^CD19^+^ (B cells). There was no difference in frequencies (not included), nor expression of MHC class II (left), or co-stimulatory molecules CD40 (center) or CD86 (right). B) T cell panels: Lymphocytes were gated on singlet, live cells and then on CD3^+^CD4^+^ (CD4^+^ T cells). There was no difference in activation as defined by CD44^hi^ CD62L^lo^ populations (left), no difference in proliferation as assessed by intracellular Ki-67 staining (center), nor was there a difference in production of IFNγ or IL-17 (intracellular cytokine staining, right). C) The abundance of MOG-reactive T cells was probed by stimulation with MOG or MOG-MDA, subsequent culture and performing Thymidine incorporation assays. Though there was antigen-specific proliferation, there were no significant differences between MOG and MOG-MDA immunization, nor the respective antigen re-stimulations. Notably, the abundance of MOG-reactive cells from immunized lymph nodes in significantly lower as compared to 2D2 transgenic mice. SI = Stimulation Index (relative to average). D) Stained lymphocytes obtained after antigen re-stimulation were gated on singlet, live cells and then on CD3^+^CD4^+^ (CD4^+^ T cells). Intracellular Ki-67 staining as marker for proliferation revealed no significant differences. Taken together, these results imply that there is no difference in priming T cell responses in B6 mice with either MOG or MOG-MDA. The bulk of analyzed cells displayed no differences, albeit this may mask the activation of individual cells that came from the periphery. Especially the antigen recalls established that both immunizations yield MOG-reactive T cells, and these T cells could be re-stimulated with either MOG or MOG-MDA. Compared to the transgenic 2D2 system, the abundance of specific T cells from lymph nodes presents a limiting factor. Thus enhanced availability of antigen (e.g. MOG-MDA) may not evoke an increased proliferation. Lastly, the EAE data presented in Fig. 5C displayed no differences, which supports the above data (TIFF 509 kb)

## Abbreviations

APC: Antigen presenting cell
CNS: Central nervous system
MAA: Malondialdehyde-acetaldehyde adduct
MDA: Malondialdehyde
MERTK: Tyrosine protein kinase MER
PAMPs/DAMPs: Pathogen/danger associated molecular patterns
SRA: Scavenger receptor A
TLR: Toll-like receptor
