# Supplementary figures and images for: Scavenger Receptor A Mediates the Clearance and Immunological Screening of MDA-Modified Antigen by M2-Type Macrophages
Source: Neuromolecular Med. 2017 Aug 21;19(4):463–79. doi: 10.1007/s12017-017-8461-y (PMC5683054; doi:10.1007/s12017-017-8461-y)

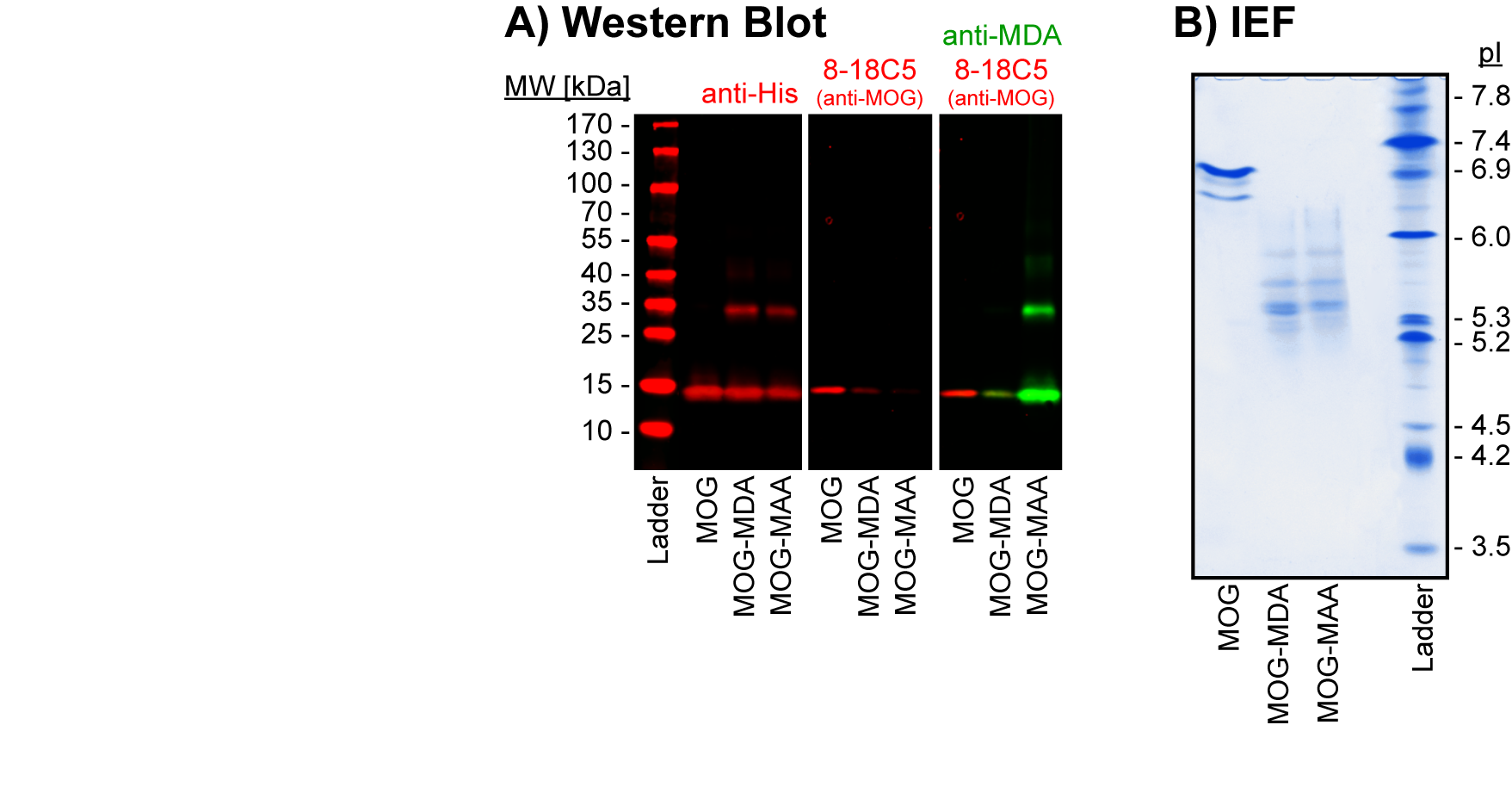

Supplement: Supplementary file 1 — Biochemical analysis of MOG and its modified variants using the MDA or MAA protocols. Recombinant mouse Myelin Oligodendrocyte Glycoprotein was purified and modified with MDA, or MDA with addition of acetaldehyde (MAA), as described in the Methods. A) Analysis by Western Blotting: The proteins can be detected using anti HIS-tag antibodies. Some crosslinking by MDA occurs as evident by the dimeric band at ~35 kDa. Notably, the recognition of MOG-MDA or MOG-MAA by the anti-MOG monoclonal 8-18C5 antibody is reduced. This antibody is conformational, but also the modified protein may bind differently to the nitrocellulose membrane. Importantly, the recognition by MDA antiserum is stronger for MOG-MAA than for MOG-MDA, implying that the generation of the recognized epitope is enhanced, presumably the MAA adduct. Similar results were obtained using the 1F83 anti-MAA monoclonal (not included). B) Analysis by isoelectric focusing: Proteins were focused according to isoelectric point (pI) as described and stained with Coomassie Blue. The modified variants display a drop in pI, indicating an increase in net negative change (loss of positive charge). The pattern between MOG-MDA and MOG-MAA is similar. In this article we used MOG modified according to the MAA protocol with addition of acetaldehyde, but refer to it as MOG-MDA to respect the fact that the chemical reaction in vitro yields a spectrum of MDA-dependent adducts (TIFF 588 kb) [file 12017_2017_8461_MOESM1_ESM.tif]

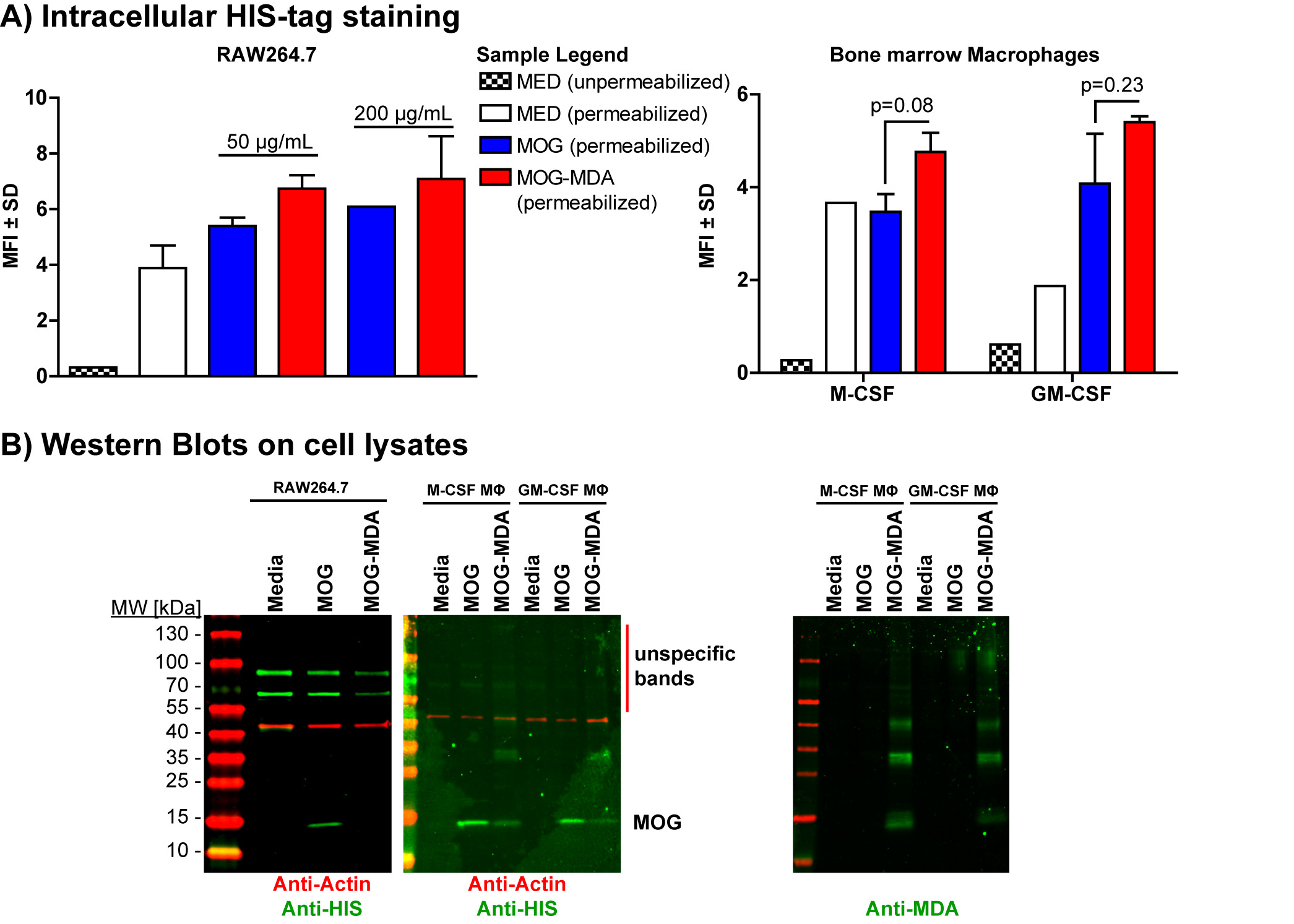

Supplement: Supplementary file 2 — Intracellular HIS-tag staining cannot be used to accurately study protein uptake. RAW264.7 cells or primary bone marrow-derived macrophages (BMMs) from C57BL/6 mice differentiated with either M-CSF or GM-CSF were cultures as described. The cells were incubated with recombinant (HIS-tagged) MOG or MOG-MDA (50 µg/mL or as indicated) for 4h. Cells were either prepared with intracellular FACS staining using or used to obtain lysates that were analyzed by Western Blotting using the indicated antibodies. A) FACS analysis: Left: RAW264.7, Right: primary bone marrow-derived macrophages. There was a profound background revealed for permeabilized cells, even in the absence of HIS-tagged MOG. The method failed to reliably detect the uptake of HIS-tagged MOG as the differences to media samples were mostly insignificant. Furthermore, there was no apparent effect with increased doses or MDA-modified MOG. B) Analysis by Western Blotting: The purified anti HIS-tag antibody was the same clone as the one used for FACS. Western Blotting revealed clear off-target staining of endogenous antigens (>70 kDa), both in RAW264.7 cells and in primary BMMs. The separation by SDS-PAGE allowed discrimination of bands corresponding to MOG at 15 kDa. The band for MOG-MDA appears to be weaker, which contradicts the observations from uptake experiments performed using the fluorescently labeled protein (cf. Figs. 1, 2). Importantly, the detection of unlabeled MOG uptake using Western Blotting is hampered by the immediate proteolytic digestion, as demonstrated in Fig. 4 of the main article. Taken together, these results establish that intracellular HIS-tag staining cannot be used to study uptake in these cells due to the background given by endogenous, intracellular targets and the inefficiency of the method to reliably detect phagocytosed antigen, along with proteolysis of phagocytosed antigen (TIFF 788 kb) [file 12017_2017_8461_MOESM2_ESM.tif]

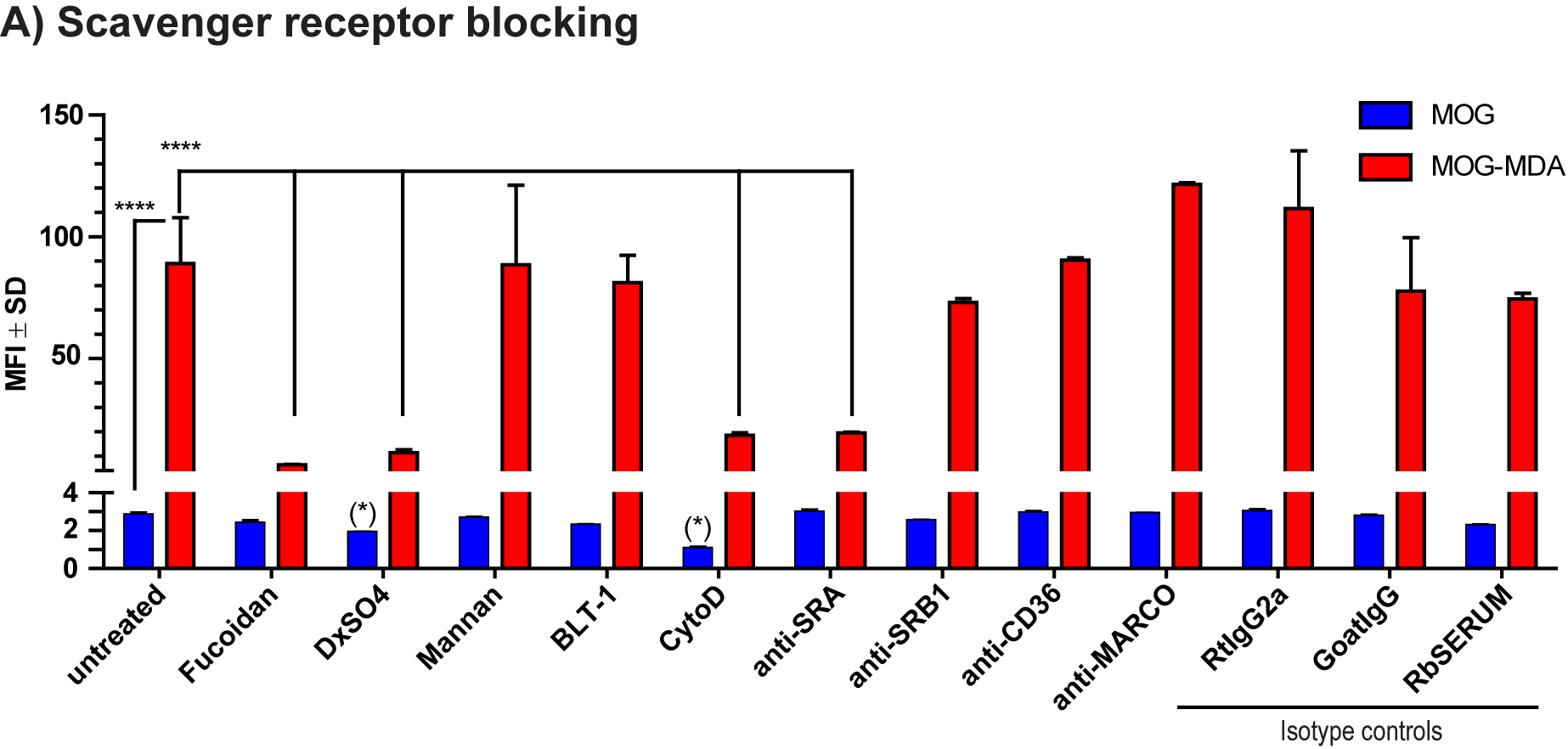

Supplement: Supplementary file 3 — Full panel of uptake inhibitors and blocking antibodies and isotype controls. This panel supports Fig. 2B of the main article, but includes samples that did not show any effect, i.e., Mannan, BLT1, anti-SRB1, anti-CD36, anti-MARCO (SCARA2), and various isotype controls. For further details we refer to the main article (TIFF 255 kb) [file 12017_2017_8461_MOESM3_ESM.tif]

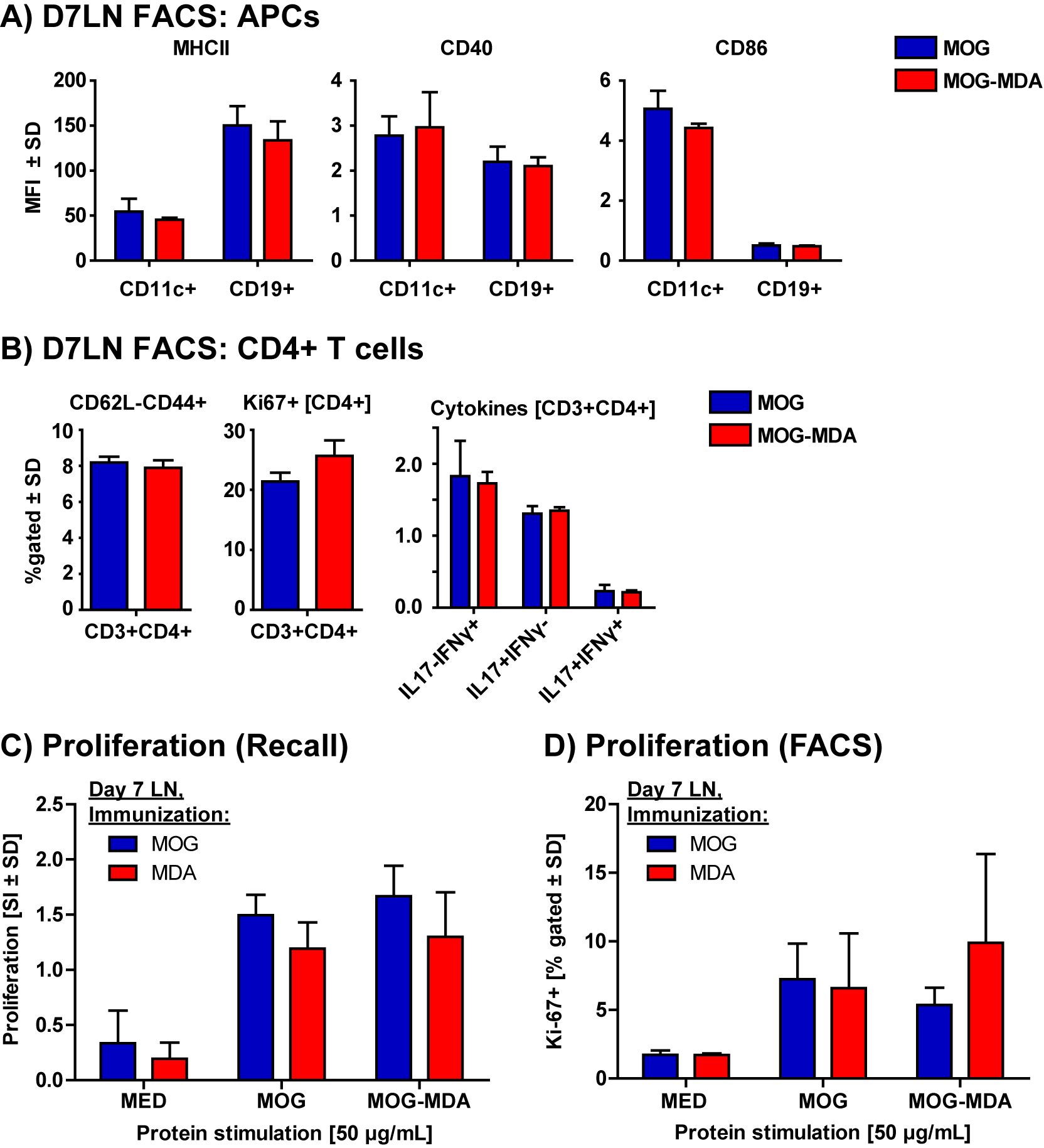

Supplement: Supplementary file 4 — FACS analysis of day 7 lymph nodes and proliferation. Day 7 lymph nodes (D7LN) from EAE-immunized B6 mice were collected and cells analyzed by flow cytometry, or incubated with 50 µg/mL of antigen for either thymidine incorporation assays or FACS analysis after recall, as described. A) APC panel: Lymphocytes were gated on singlet, live cells and then on CD11c+CD19− (CD11c+ APCs) or CD11c−CD19+ (B cells). There was no difference in frequencies (not included), nor expression of MHC class II (left), or co-stimulatory molecules CD40 (center) or CD86 (right). B) T cell panels: Lymphocytes were gated on singlet, live cells and then on CD3+CD4+ (CD4+ T cells). There was no difference in activation as defined by CD44hi CD62Llo populations (left), no difference in proliferation as assessed by intracellular Ki-67 staining (center), nor was there a difference in production of IFNγ or IL-17 (intracellular cytokine staining, right). C) The abundance of MOG-reactive T cells was probed by stimulation with MOG or MOG-MDA, subsequent culture and performing Thymidine incorporation assays. Though there was antigen-specific proliferation, there were no significant differences between MOG and MOG-MDA immunization, nor the respective antigen re-stimulations. Notably, the abundance of MOG-reactive cells from immunized lymph nodes in significantly lower as compared to 2D2 transgenic mice. SI = Stimulation Index (relative to average). D) Stained lymphocytes obtained after antigen re-stimulation were gated on singlet, live cells and then on CD3+CD4+ (CD4+ T cells). Intracellular Ki-67 staining as marker for proliferation revealed no significant differences. Taken together, these results imply that there is no difference in priming T cell responses in B6 mice with either MOG or MOG-MDA. The bulk of analyzed cells displayed no differences, albeit this may mask the activation of individual cells that came from the periphery. Especially the antigen recalls established that both immunizations yie [file 12017_2017_8461_MOESM4_ESM.tif]
